# Modeling policy decisions to mitigate the risk of emerging arboviral diseases under ecological changes in Uganda: Proposing a one Health in all policies approach

**DOI:** 10.1016/j.onehlt.2026.101414

**Published:** 2026-04-17

**Authors:** Maureen Nabatanzi, Selina L. Graff, Kigai E.E. Bigala, Peter Z. Sabakaki, Teddy A. Tindyebwa, Julius J. Lutwama, Innocent B. Rwego, Anthony M. Nsubuga, Sandra Junglen, Lisa Biber-Freudenberger

**Affiliations:** a Center for Development Research (ZEF), University of Bonn, Germany; b Institute of Virology Charité - Universitätsmedizin, Berlin, Germany; c College of Veterinary Medicine, Animal Resources and Bio-security, Makerere University, Kampala, Uganda; d Department of Plant Sciences, Microbiology and Biotechnology, Makerere University, Kampala, Uganda; e Uganda Virus Research Institute, Entebbe, Uganda

**Keywords:** Arbovirus, Vector-borne disease, One Health, Biodiversity, Climate change, Policy, Decision, Model

## Abstract

Land-use and ecological changes are major drivers of emerging arboviral diseases. Uganda, a biodiversity-rich country with high vulnerability to arboviruses, is experiencing intensifying ecological pressures. However, the potential of different policies to reduce the impact of ecological disruptions on arboviral risk remains insufficiently understood. In this study, we combined policy review and expert opinion elicitation within a Structured Decision Modeling framework to evaluate how different policy interventions may reduce arboviral disease risk in Uganda. During two workshops, 20 multidisciplinary experts reviewed existing policies and identified disease preventive interventions. Experts assessed the influence of 29 preventive actions on disease risk under four policy scenarios: 1) Do nothing (DN), 2) Increase biodiversity conservation (B), 3) Implement human and animal health measures (H), and 4) apply a One Health approach (OH)– combining biodiversity conservation and health measures. The OH scenario produced the highest probability of low arboviral risk (82%), compared to H (16%), B (14%), and DN (0.006%). Under OH, mosquito net use, vector site removal, and multi-level surveillance actions had high probabilities of implementation (>80%). When actions were clustered, the probability of implementation was highest for biodiversity protection actions (72%), compared to arbovirus surveillance actions (69%), interdisciplinary actions (59%), and vector control actions (55%). Mutual Information analysis identified interdisciplinary actions as most beneficial for reducing decision uncertainty. The OH scenario had the highest Expected Value of Perfect Information, indicating a 25% improvement in decision confidence given perfect evidence. This study demonstrates the added benefit of integrating socio-ecological and human health dimensions that were separately less effective. This calls for embedding One Health in All Policies to improve OH governance. We present a structured, participatory framework to prioritise multi-sectoral preventive actions and improve collaborative implementation. This strategic approach is pragmatic for arboviral threats in complex, ecologically sensitive settings like Uganda.

## 1 Introduction

The global surge in (re)emerging infectious diseases is increasingly driven by complex ecological and epidemiological interactions [1]. Arthropod-borne viruses (arboviruses), transmitted by infected arthropods such as mosquitoes, sand flies, or ticks, are zoonoses implicated in more than 80 human diseases [2]. In 2023, the estimated global burden in Disability-Adjusted Life Years per 100,000 population was 290,137 (107,073–597,713) for Yellow fever, and 6156 (353–11,958) for Rift Valley Fever (RVF) [3]. In Africa, where many arboviral diseases first emerge, they present a growing public health threat [4]. For example, an outbreak of Yellow fever between 2015 and 2016 in Angola and the Democratic Republic of Congo affected 7334 people with 393 deaths [5]. A resurgence of the disease is ongoing; at least 248 people across 17 African countries were affected in 2024 alone [6]. Moreover, unlike RVF and Yellow fever, which have animal and human vaccines, respectively, West Nile fever still has no vaccine, yet it is endemic in Africa, has been detected in multiple species, and has caused outbreaks in the Democratic Republic of Congo (DRC) and Sudan, and epizootics in South Africa [7]. In addition, the diagnosis and control of arboviral diseases are constrained by the common presentation of non-specific febrile symptoms and the need for advanced laboratory confirmatory tests [7].

Uganda, where *Zika*, *West Nile*, *Semuliki Forest*, and *O'nyong-nyong viruses* were first detected [8], experiences recurrent outbreaks. During 2000–2020, Uganda reported 16 RVF, and six Yellow fever outbreaks (with 45 deaths in 2010 and 14 deaths in 2018) [[9], [10], [11]]. However, detection of other likely endemic and (re)emerging arboviral diseases like Wesselsbron fever and Sindbis fever is undermined by co-circulation with malaria, RVF, and Yellow fever, limited diagnostics, and exclusion from the Integrated Disease Surveillance and Response (IDSR) framework– the backbone of surveillance [12,13]. Furthermore, Uganda's tropical ecosystems are increasingly threatened by climate change, deforestation, wildlife exploitation, and rapid expansion in agriculture and infrastructure [14]. Despite these vulnerabilities, surveillance and response efforts are heavily skewed towards human health, overlooking the ecological and multi-species transmission dynamics [15].

The ecological predictors of arboviral disease emergence necessitate One Health (OH)— an integrated approach involving coordinated efforts by specialists of human, animal, environmental health and other disciplines [10,16,17]. Although desirable, operationalisation of OH in Africa is still siloed in institutional programs, constraining integration and coordination [[17], [18], [19]]. Fundamentally, the institutionalization of OH is constrained by inadequate policy and legal frameworks and persistent challenges in implementing multisectoral health policies [20,21]. This cascades into conflicts over governmental/sectoral mandates, which have inhibited effective collaboration in Nigeria, Uganda, and Guinea [20]. Additional barriers to OH implementation in Africa include focus on topic-specific interventions, inadequate awareness among stakeholders, limited surveillance capacity, and insufficient financial and human resources [[20], [21], [22], [23]]. Despite this, the recurrence of zoonotic disease threats, political will, including national OH platforms as well as global health security commitments, are recognized as catalysts fostering OH initiatives in Burkina Faso, Guinea, the Democratic Republic of Congo, and Uganda [20,21,23].

Uganda has adopted several health security frameworks, including the International Health Regulations (2005), Integrated Disease Surveillance and Response (IDSR) [12,24], and a One Health Strategic Plan [15]. However, weak intersectoral coordination—especially between human and veterinary health as well as environmental sectors—limits effective zoonotic disease control [15,23].

Decision-making on disease prevention becomes more complex due to limited resources, competing policy priorities, and uncertainties like gaps in epidemiological and ecological data [25,26]. While various models exist to assist public health decision-making [27,28], recent approaches emphasise fit-for-purpose models that align with decision-makers' goals and facilitate actionable trade-offs [25,26,29]. Structured Decision Making (SDM) provides a framework that supports stakeholder involvement and transparent evaluation of policy alternatives under uncertainty [30]. It has been used to inform decision-making in agriculture, environment, and infectious diseases, and is well-suited for OH challenges requiring stakeholder alignment [26,31,32]. In this study, we integrate stakeholder engagement in SDM to explore policy options for reducing arboviral disease risk in Uganda. We hypothesize that policy strategies integrating actions across multiple One Health sectors will yield more favorable risk outcomes than fragmented approaches, and that a Bayesian decision analysis framework can provide a transparent and quantitative basis for comparing these policy options under uncertainty. To our knowledge, this is the first participatory modeling effort to quantify the relative impact of biodiversity, health, and integrated OH policies on arboviral risk in Uganda.

## 2 Materials and methods

### 2.1 Study site

Uganda, located between 4^o^N – 1^o^S and 29.5^o^W – 35^o^W, is a biodiversity hotspot encompassing diverse ecosystems, including the Albertine Rift, montane forests, savannahs, and wetlands [14]. Uganda frequently reports multiple arboviral disease outbreaks, with challenges in timely prevention [[8], [9], [10]].

### 2.2 Structured decision modeling

Structured Decision Modeling (SDM) is used to integrate expert knowledge, clarify causal relationships, and support decision-making under uncertainty. [31,33]. The SDM process followed the “PrOACT” framework (Problem, Objectives, Alternatives, Consequences, Trade-offs) [34], see Supplementary Text 1.

#### 2.2.1 Problem framing and evidence synthesis

We conducted a policy analysis to identify strategies relevant to reducing arboviral risk in Uganda [35]. We performed a targeted search of official government and institutional websites (e.g., ministries responsible for health, agriculture, environment, and wildlife) to identify publicly available policy and strategy documents. Search terms included combinations of “diseases”, “health”, “climate change”, “wildlife”, “biodiversity”, “land use”, “environment”, “policy”, “guideline”, “strategy”. We screened the documents for relevance based on their thematic connection to arboviral risk and One Health domains. This process resulted in the inclusion of 13 key policy documents. We extracted disease preventive interventions, which we collated into thematic clusters.

#### 2.2.2 Identification of decision objectives

We purposively invited 20 experts from government, academia, and civil society across health, agriculture, environment, and wildlife sectors (see Supplementary Table 1). The stakeholders were selected based on their knowledge, diversity, involvement in decision making experience working at both national and sub-national levels. [31,36,37]. Through two workshops, experts validated the policy analysis, identified additional policies, and defined four policy scenarios for disease mitigation. These scenarios represent the decision alternatives evaluated in the model [38].

#### 2.2.3 Specification of alternative actions and valuation of their impacts

**Fig. 1:**
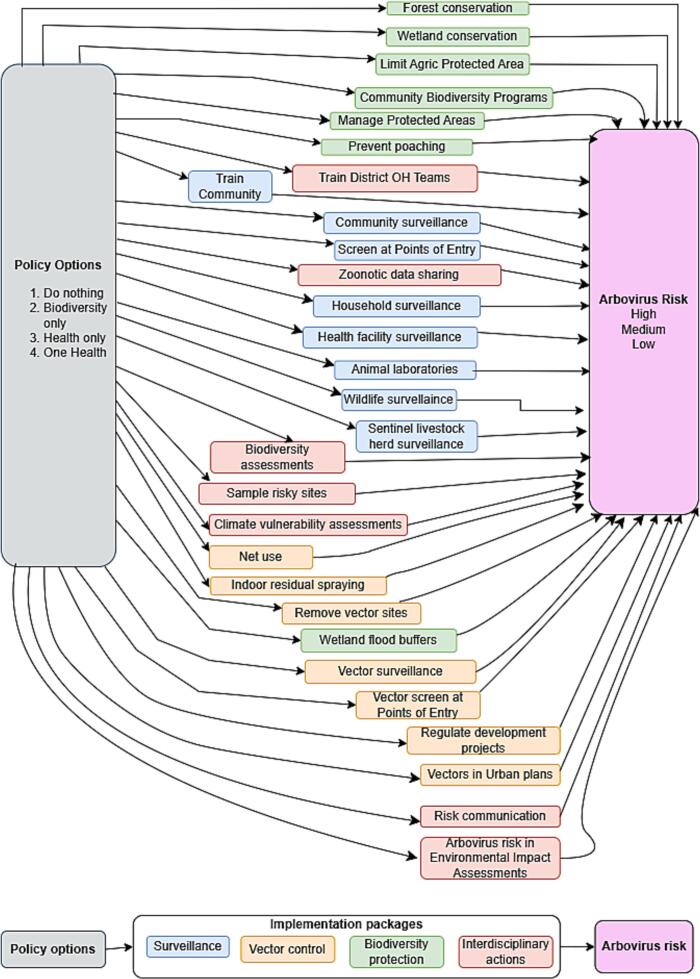
Directed acyclic graph (DAG) linking policy options, implementation packages, and arbovirus risk. Policy options are indicated in grey, preventive actions are colour-coded according to their implementation package, and arbovirus risk is indicated in purple. The DAG was produced using draw.io [40]. (For interpretation of the references to colour in this figure legend, the reader is referred to the web version of this article.)

Through participatory discussions (See Supplementary Text 1) and using a schematic summary of the policy analysis, stakeholders reached consensus on 29 preventive actions [39]. These were clustered into four implementation packages: surveillance, biodiversity protection, vector control, and interdisciplinary actions. These relationships were represented in a directed acyclic graph (DAG) linking policy options, implementation packages, and disease risk (Fig. 1) [38].

#### 2.2.4 Evaluation of outcomes using Bayesian models

Bayesian decision modeling in SDM applies Bayes' theorem to incorporate expert knowledge in probabilistic models [39]. We transformed the DAG into a Bayesian Network (BN), where policy options (parent nodes) influence preventive actions (child nodes) which in turn affect implementation packages and overall arboviral risk. Experts underwent a calibration exercise to improve their ability to express uncertainty and reduce estimation biases (see Supplementary Text 1) [36,38]. Experts then estimated conditional probabilities for each preventive action given policy scenarios [38,41].

We assumed that better implementation of actions in combination would collectively improve the likelihood of the implementation packages and reduce arboviral risk. We then applied weighted scoring to compute conditional probabilities for the implementation package and arboviral risk nodes [38,39], as described in Supplementary Text 1 (Eqs. 3–5). The BN was implemented in Netica version 7.01 [42]. The policy summary diagram was produced using draw.io [40].

#### 2.2.5 Trade-off analysis to optimize decisions

We performed sensitivity analysis to assess how variation in each of the probabilities of implementation packages and their combinations influenced arboviral risk across policy scenarios [43,44]. We plotted the results as a heat map in R using ggplot2 [45].

The Value of Information (VoI) analysis resolves some uncertainty by identifying those variables for which more information would maximise the model output and clarify the decision [26,36,38]. We calculated the Expected Monetary Value (EMV), Expected Value with Perfect Information (EV with PI), and Expected Value of Perfect Information (EVPI) to determine which policy provided the highest expected utility under uncertainty. Detailed computation steps are provided in Supplementary Text 1 (Eqs. 3–5)*.* We visualized the VoI analysis result in R using ggplot2 [45].

## 3 Results

We applied structured decision-making (SDM) to integrate multidisciplinary knowledge and predict effective policy options for mitigating arboviral disease risk in Uganda. The process involved a systematic policy analysis, stakeholder-informed problem framing, and Bayesian simulation of alternative disease preventive policy scenarios.

### 3.1 Policy analysis

We reviewed 19 national policy documents relevant to arboviral diseases from the Ministries of Health (MoH) (4), Agriculture, Animal Industry and Fisheries (MAAIF) (5), Water and Environment (MWE) (5), and Tourism, Wildlife, and Antiquities (2) as well as multisectoral frameworks (3) (see Supplementary Table 2). We identified strong ambitions for multi-sectoral collaboration in disease, environment and wildlife management, policy co-development and as intentions to co-implement, particularly, in the multisectoral frameworks.

The MoH strategic plan promoted a “Health in All Policies” approach requiring non-health sectoral policies to promote health. One Health was a foundational principle in the MoH, the multisectoral policies and in the infection prevention policy of the MAAIF. However, environment and wildlife policies placed limited emphasis on OH. Ecological and wildlife determinants of zoonoses were scanty across all policies. Surveillance guidance largely focused on human case detection with limited consideration of the sentinel, animal, environmental and genomic components. Inter-sectoral implementation and funding responsibilities for cross-cutting activities like risk assessments were unclear.

**Fig. 2:**
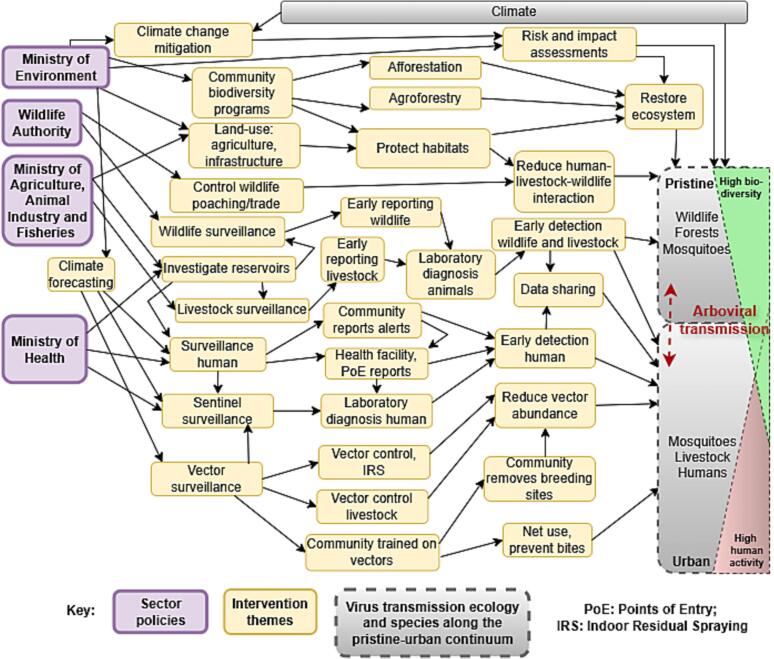
Schematic summary of the policy analysis. In purple are the main sectors whose policies informed the selection of themes (in yellow) that play a role in preventing arboviral disease transmission in diverse ecosystems (in grey, red and green). (For interpretation of the references to colour in this figure legend, the reader is referred to the web version of this article.)

We identified 162 preventive interventions (see Supplementary Table 2), which we collated into 67 thematic implementation packages (see Supplementary Table 3). We summarised these themes in a schematic diagram to guide the stakeholders' selection of priority actions (Fig. 2). The diagram shows the four main Ministries whose policies guide the majority of One Health thematic interventions, whose implementation and interaction in turn can contribute to mitigating the potential risk of arboviral disease transmission in ecosystems. Diversity in ecosystems is represented by pristine regions characterised by higher biodiversity, and increasingly urban regions. There is a porous interface connecting the two ecological regions where mosquitoes, wildlife, forests, livestock, and humans can traverse and interact with implications for transmission risk.

### 3.2 Problem framing and objective setting

Based on the policy analysis and workshops, we framed the decision problem as identifying the most effective policy interventions for reducing arboviral disease transmission under varying socio-ecological and epidemiological conditions. This need stems from Uganda's vulnerability to zoonotic outbreaks coupled with ecological disturbances and resource constraints. Stakeholders examined the 67 themes, prioritized 29 preventive actions, and grouped them into four implementation packages: surveillance, biodiversity protection, vector control, and interdisciplinary actions (Supplementary Table 4). The overarching goal was to minimize arboviral risk.

### 3.3 Policy option determination

During the workshops, stakeholders proposed four policy scenarios:

1. Do Nothing (DN): Counterfactual scenario with no specific preventive interventions.
2. Biodiversity conservation (B): Focused on preserving ecological integrity, improving equitable community benefits from conservation and environmental safeguards for infrastructure development. Forest and wetland destruction, illegal wildlife poaching and agriculture in protected areas were minimised.
3. Human and Animal Health (H): Prioritized disease prevention and early detection through human, livestock and wildlife health monitoring and community engagement. Cross-species disease transmission was minimised and vector control implemented.
4. One Health (OH): A holistic approach combining biodiversity conservation and health interventions. Environmental protection efforts, as well as all relevant sectors, sub-national and community players, were actively integrated in disease prevention, detection and response.

### 3.4 Policy scenario outcomes

#### 3.4.1 Do nothing

**Supplementary Fig. 1:**
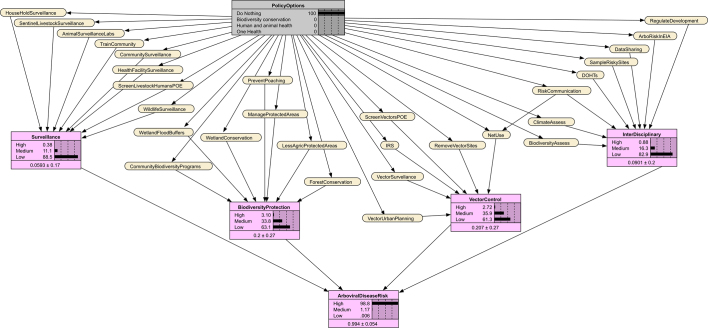
Bayesian Network for the Do Nothing scenario.

None of the 29 preventive actions analysed exceeded a 40% probability of implementation, resulting in the highest likelihood of arboviral disease risk (99%) among all scenarios (Table 1). Supplementary Fig. 1 shows the Bayesian Network for the Do Nothing scenario.

#### 3.4.2 Biodiversity conservation

**Supplementary Fig. 2:**
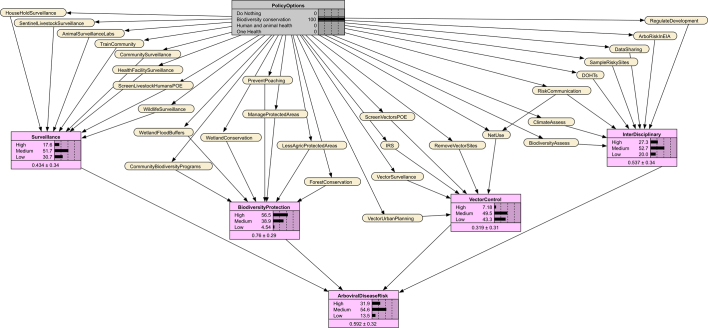
Bayesian Network for the Biodiversity Conservation scenario.

Fourteen (48%) preventive actions had at least a 60% probability of implementation, led by poaching prevention, biodiversity assessments, and wetland conservation (Table 1). Probabilities for the implementation packages and risk of arboviral disease transmission risk was as all medium (54%). Supplementary Fig. 2 shows the Bayesian Network for the Biodiversity conservation scenario.

#### 3.4.3 Human and animal health

**Supplementary Fig. 3:**
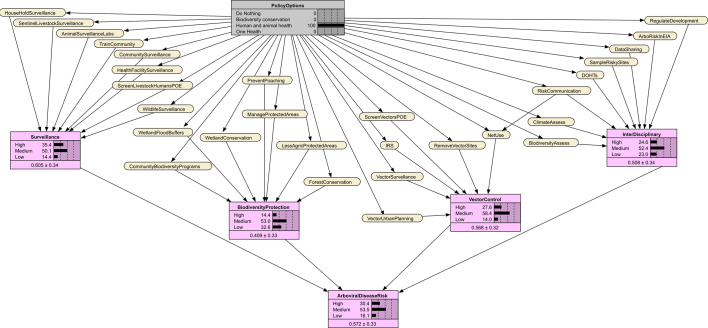
Bayesian Network for the Human and Animal Health scenario.

Sixteen preventive actions (55%) had implementation probabilities ≥60%, led by mosquito net use, health facility surveillance, and expansion of regional animal health surveillance laboratories and sites (Table 1). Probabilities were moderate across all implementation packages, resulting in a medium likelihood of risk for arboviral disease transmission (54%). Supplementary Fig. 3 shows the Bayesian Network for the Human and animal health scenario.

#### 3.4.4 One Health

**Fig. 3:**
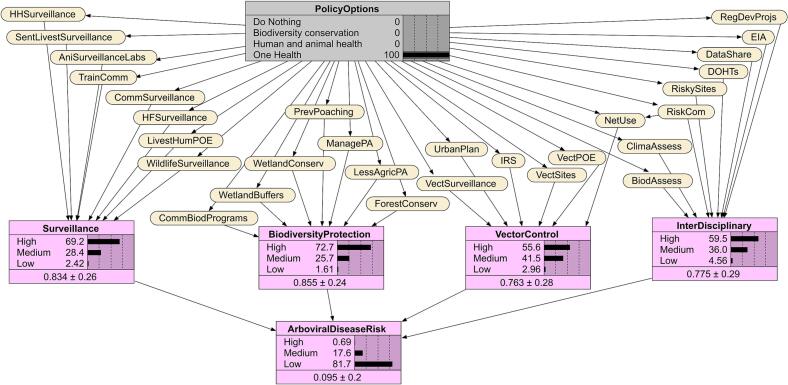
Bayesian Network of predicted impact pathways under the One Health policy scenario. The grey box indicates the One Health scenario, in yellow are the preventive actions and in purple are the percentage probabilities of the implementation packages and arboviral disease risk. Abbreviations of preventive actions are defined in Table 1. (For interpretation of the references to colour in this figure legend, the reader is referred to the web version of this article.)

All 29 preventive actions exceeded a 70% implementation probability (Table 1). The most likely were mosquito net use, vector site removal, wetland conservation, animal health laboratories, and multi-level surveillance. The Bayesian Network for the One Health scenario indicates the probabilities of preventive actions, implementation packages, and arboviral disease risk given this scenario (Fig. 3). Probabilities for the implementation packages were strongest for biodiversity (72%), and surveillance (69%), resulting in the highest likelihood of low risk of arboviral disease transmission (82%).

**Table 1:** Node probabilities of implementation under different policy options in the Bayesian Network.

Nodes	Abbreviation in BN	Do Nothing	Biodiversity conservation	Human and animal health	One Health
Preventive action		% Probability for State = Yes	% Probability for State = Yes	% Probability for State = Yes	% Probability for State = Yes
Train the community and farmers to identify and report	TrainComm	20	50	60	70
Surveillance by community health, wildlife and extension workers	CommSurveillance	10	55	55	80
Screen livestock and humans at Points of Entry (PoE)	LivestHumPOE	23	50	65	74
Conduct surveillance among high-risk households	HHSurveillance	30	40	60	70
Health facility sentinel surveillance	HFSurveillance	35	45	70	80
Regional animal health surveillance laboratories and sites	AniSurveillanceLabs	30	50	70	80
Wildlife surveillance	WildlifeSurveillance	30	65	55	80
Sentinel livestock herd surveillance	SentLivestSurveillance	25	65	60	70
Forest conservation	ForestConserv	35	60	36	75
Wetland conservation	WetlandConserv	30	70	55	80
Limit agriculture in protected areas	LessAgricPA	30	68	45	70
Community wildlife biodiversity programs	CommBiodPrograms	30	70	40	75
Manage protected areas	ManagePA	25	63	40	70
Prevent wildlife poaching	PrevPoaching	25	75	50	75
Wetlands as floodwater buffers	WetlandBuffers	40	60	45	70
Mosquito net use	NetUse	40	45	77	91
Indoor residual spraying	IRS	40	45	65	70
Remove vector breeding sites	VectSites	30	45	60	80
Vector surveillance	VectSurveillance	35	45	60	70
Vector screening at PoE	VectPOE	35	43	60	70
Vector-sensitive urban planning	UrbanPlan	30	50	55	75
Train District One Health Teams	DOHTs	35	40	62.5	74
Intersectoral zoonotic and climate data sharing	DataShare	25	60	60	70
Biodiversity assessments at risky sites	BiodAssess	30	70	55	75
Mosquito animal and human sampling at risky sites	RiskySites	25	55	60	70
Climate vulnerability assessment	ClimaAssess	30	65	50	70
Regulate infrastructural and mineral projects	RegDevProjs	40	65	45	75
Risk communication	RiskCom	30	50	60	70
Include arbovirus risk in environmental impact assessments	EIA	25	60	60	70
Nodes	State	Do nothing	Biodiversity conservation	Human and animal health	One Health
Implementation package		% Probability	% Probability	% Probability	% Probability
Surveillance	High	0.38	18	35	69
	Medium	11	52	50	28
	Low	89	31	14	2.4
Biodiversity protection	High	3.1	57	14	73
	Medium	34	39	53	26
	Low	63	4.5	33	1.6
Vector control	High	2.7	7.2	28	56
	Medium	36	50	58	41
	Low	61	43	14	3.0
Interdisciplinary action	High	0.88	27	25	59
	Medium	16	53	52	36
	Low	83	20	35	4.6
Decision outcome					
Arboviral disease risk	High	99	32	30	0
	Medium	1	54	54	18
	Low	0	14	16	82

### 3.5 Sensitivity and VoI findings

**Fig. 4:**
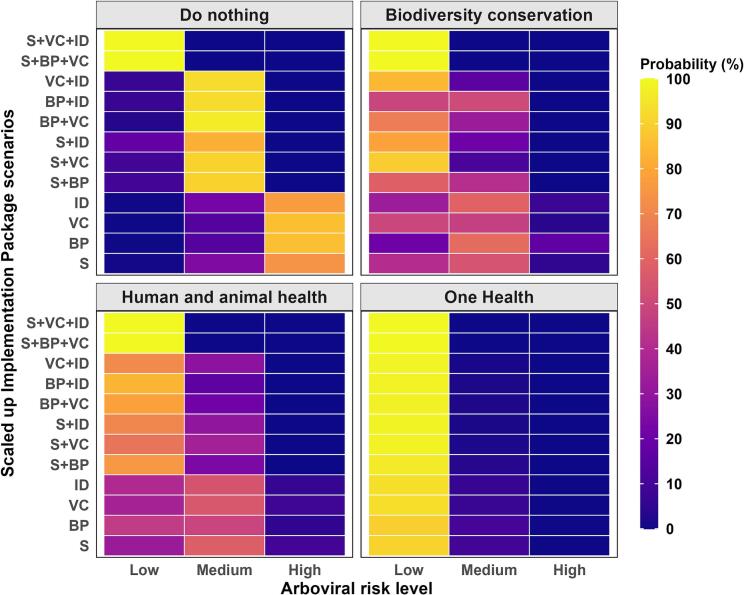
Percentage probability of arboviral disease risk under alternate policies given that the scenario states “High” of the implementation packages are scaled up to 100% singly or in combination. Abbreviations for Implementation packages: S = Surveillance, BP = Biodiversity protection, VC = Vector control and ID = Interdisciplinary. + indicates a combination. Probabilities increase from darker colours towards yellow. (For interpretation of the references to colour in this figure legend, the reader is referred to the web version of this article.)

The sensitivity analysis heat map indicated that when the implementation packages were scaled up, meaning the scenario state “High” for that package was 100%, the OH scenario consistently showed the highest probability of low arboviral risk (depicted in yellow) (Fig. 4). For OH, the most significant individual impacts were from interdisciplinary actions (94%) and vector control (93%); combined, they resulted in a 99% chance of low arboviral risk. Under OH, increasing any single package always reduced arboviral risk to low. In all scenarios, scaling up any three groups shifted arboviral risk to low (Fig. 4). For details, see Supplementary Table 5.

Mutual Information (MI) assessed how much knowing the state of each implementation package reduced uncertainty about the risk of arboviral disease transmission. Under OH, MI was greatest for interdisciplinary actions (17%) (Table 2).

**Table 2:** Analysis of sensitivity of arboviral disease risk to findings per preventive group.

Sensitivity of risk to a preventive group node reduction in uncertainty	One Health		Human and animal health		Biodiversity conservation		Do nothing	
	Mutual Information	Percent	Mutual Information	Percent	Mutual Information	Percent	Mutual Information	Percent
Interdisciplinary	0.126	17.2	0.195	13.6	0.193	13.9	0.016	17.1
Vector control	0.102	14	0.158	11.1	0.153	10.9	0.016	17.7
Surveillance	0.093	12.8	0.169	11.8	0.196	14.1	0.012	12.7
Biodiversity protection	0.082	11.2	0.165	11.5	0.129	9.28	0.018	19.4

**Fig. 5:**
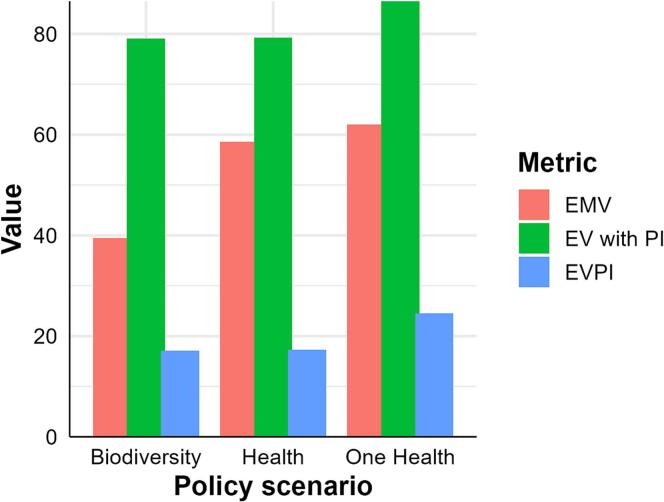
Value of Information analysis comparing the Expected Monetary Value (EMV), Expected Value with Perfect Information (EV with PI), and Expected Value of Perfect Information (EVPI) across policy scenarios. For the policy scenarios, Biodiversity = Biodiversity Conservation, Health = Human and Animal Health, and One Health.

The OH scenario provided the best decision utility, with an Expected Monetary Value (EMV) of 62%, Expected Value with Perfect Information (EV with PI) of 86%, and Expected Value of Perfect Information (EVPI) of 25%. While scenario H had a higher EMV than B (58% vs. 39%), both showed similar EV with PI and EVPI values (Fig. 5, Supplementary Table 6). In Fig. 5, the difference between EMV and EV with PI represents the potential value of reducing uncertainty, with higher EVPI indicating greater benefit from additional information.

## 4 Discussion and conclusion

This study applied expert-informed SDM to evaluate policy options for arboviral disease prevention in Uganda. Findings highlight that integrating biodiversity conservation with human and animal health—through a OH approach—offers the greatest potential for reducing arboviral risk. A key strength of this study is its participatory design, which engaged stakeholders to co-develop realistic actionable intervention scenarios.

### 4.1 Policy implications

Policy review revealed a strong intent for multisectoral collaboration, but with limited operationalisation across agricultural and environmental sectors. Although the “Health in All Policies” principle was emphasized in several frameworks, ecological and wildlife determinants of disease remain weakly addressed. Financing and coordination for cross-sectoral activities, such as risk assessments were often undefined.

Results identified the OH scenario as the most effective, followed by Human and Animal Health (H) and Biodiversity Conservation (B), while the Do Nothing scenario (DN) was ineffective. These findings reinforce the global endorsement of OH for pandemic prevention, preparedness and response under the WHO Pandemic Agreement [17,18,46]. Since arboviral diseases emerge in pristine environments, OH interventions that address environmental drivers of emergence and transmission are crucial [4,19]. In the African context, this necessitates policy and legal frameworks that transcend governmental mandates and institutional silos to bridge collaborative preventive actions across human, animal, and environmental health [20,21]. Although multiple African governments, including Nigeria, Tanzania, Uganda, and Kenya, have demonstrated some political will concerning OH, for example, through strategic policies and national coordination platforms, their impact is still constrained by inadequate human, material and financial resources from the government [18,20]. In addition, uneven representation of resource and technical capacities leads to an under-representation of actors from wildlife, environment, and the social sciences [18]. In Uganda's case, persistent policy gaps, limited integration of non-human and animal health sectors, and gaps in sustainable funding are some of the major challenges to OH advancement [12,14,23,47]. The 2025 Lancet OH Commission proposes interconnected systems that go beyond disease surveillance to include climate change mitigation, conservation, and the whole society [19]. Given the high mutual information ranking, Uganda could optimize resources by strengthening interdisciplinary actions and vector control. Expanding the “Health in All Policies” concept to a “One Health in All Policies” framework would promote active engagement of non-health sectors [18,19]. Institutionalizing OH with measurable outcomes, sustainable funding, and operational frameworks could improve implementation and accountability [17,18,24]. A 2025 review identified key drivers of OH implementation, including research and stakeholder engagement in countries such as Nigeria, Tanzania, and Uganda, as well as increased awareness and local advocacy in Burkina Faso and Tanzania [20]. Indeed enhancing leadership awareness and communication at all levels of society would further support its integration into governance and policy [19].

### 4.2 Prioritizing preventive actions

The OH scenario had the strongest likelihood of implementing preventive actions, notably, for mosquito net use, vector site removal, wetland conservation, and multi-level surveillance. The joint scaling of interdisciplinary and vector control packages produced the largest risk reduction. Interdisciplinary measures—such as integrating ecological and agricultural data into risk assessments, data sharing, and strengthening district OH teams—can improve early detection and response [17,23]. Establishing interoperable health information systems that incorporate environmental indicators would enhance early warning [17]. Environmental impact assessments (EIA) that account for climate change and disease risk could improve ecological risk mitigation, particularly in arboviral-prone ecosystems like the Albertine Rift, which is facing threats from oil and mining activities [48,49].

Vector control showed high implementation likelihood for mosquito net use and vector site removal. Integrated vector control approaches can be complemented by safe and context-appropriate measures like repellents, traps, and biological agents [50]. Leveraging existing programs—such as mosquito net distribution and indoor residual spraying—while engaging local leaders and urban planners can reduce vector breeding in expanding towns [51].

Surveillance priorities included regional animal health laboratories and multi-level surveillance linking human, animal, and environmental data. Timely detection of cross-species transmission events necessitates collaborative surveillance [17]. Leveraging IDSR to link with environmental and veterinary surveillance systems is essential. Although Uganda has expanded its wildlife veterinary laboratory network, capacities at sub-national livestock laboratories are low [15]. Also, current sentinel systems are limited to humans, are syndromic, and involve few health facilities [52], underscoring the need for expanded genomic and animal-based monitoring [12,15,53,54]. Sentinel surveillance could utilize known animal reservoirs/hosts, for example, the use of sentinel pigeons for the surveillance of *Sindbis* and *West Nile viruses* in South Africa demonstrated peak infections during warm, wet seasons and *Culex univittatus* as a key vector [55]. In biodiversity protection, preventing poaching, conserving wetlands, and community biodiversity programs emerged as important. Human encroachment into wildlife habitats increases spill-over risks by bringing humans in contact with wildlife reservoirs of arboviruses [4,11]. Engaging local communities to address socioeconomic drivers and promote sustainable livelihood alternatives could strengthen wildlife stewardship and reduce the risk of zoonotic disease [47,56].

### 4.3 Investment in additional information

The OH scenario produced the highest EVPI, and interdisciplinary actions offered the greatest reduction in uncertainty about arboviral risk. This advises policymakers that prioritizing research and monitoring within interdisciplinary domains—such as operationalisation of OH, linking EIA and inter-sectoral surveillance data—would yield the greatest policy returns [26,31,32,37]. In addition, countries need frameworks that streamline the sharing of research outputs among the various OH stakeholders, which will be instrumental in their utilization in interventions [18].

### 4.4 Model evaluation

We evaluated model performance and robustness using sensitivity and Value of Information (VoI) analyses. These standard approaches in Bayesian decision modeling assess the influence of input uncertainty on model outcomes and prioritise variables for further data collection [33,36,38,44]. Sensitivity analysis enabled us to identify the most influential implementation packages driving arboviral risk, while VoI metrics quantified the potential benefit of reducing uncertainty in the scenarios [22,38,44]. These approaches are applied within SDM frameworks to support transparent and evidence-informed decision-making under uncertainty and complex circumstances [38,44,57].

## 5 Limitations

This study relied on expert judgement from a purposive sample which, while rich in multi-disciplinary insights, may not capture all perspectives. We endeavoured to manage experts' cognitive biases and variability through calibration efforts [36,38]. Despite this, future work should incorporate empirical data on intervention cost-effectiveness, longitudinal surveillance and financing. Furthermore, the policy analysis reviewed documents most relevant to arboviral prevention, and therefore, the list of preventive actions is not exhaustive. However, we highlight that even the implementation of a few suggested preventive actions in an interdisciplinary mechanism can significantly contribute to disease prevention.

Our modeling approach used simplified weighting schemes and discretised states (e.g., High, Medium, Low) which may obscure nonlinear interactions between preventive actions. In addition, the Bayesian Network structure assumes conditional independence among the implementation packages, which may not fully capture complex ecological and socio-institutional feedbacks inherent in real-world OH systems. Nonetheless, such participatory models can be applied to decision-making for exploring scenarios, structuring uncertainty, and guiding public health action prioritisation in data-limited contexts [31,32,37].

## 6 Conclusion

This study suggests the value of participatory decision analysis in integrating expert knowledge into arboviral prevention planning. Results highlight that a One Health in All Policies approach—combining health, biodiversity, and environmental actions—provides the most effective pathway to reduce arboviral disease risk in Uganda. We propose options for Uganda's policy makers to improve governance for multi-sectoral collaboration and actions for holistic prevention. Embedding OH principles into diverse sectors and implementation frameworks offers a practical strategy for low- and middle-income countries facing complex ecological and zoonotic challenges, however, their implementation requires policy support.

The following are the supplementary data related to this article.

Supplementary Text 1 Detailed methods. Supplementary Text 1

Supplementary Table 1 Anonymised list of experts who participated in the workshops. Supplementary Table 1

Supplementary Table 2 Summary of policy documents reviewed and identified intervention themes and gaps linked to arboviral disease risk mitigation. Supplementary Table 2

Supplementary Table 3 Intervention themes identified in the policy analysis and the number of times they appeared. Supplementary Table 3

Supplementary Table 4 Preventive actions and implementation packages. Supplementary Table 4

Supplementary Table 5 Percentage probability of arboviral disease risk under alternate policies given that the scenario states “High” of the implementation packages are scaled up to 100% singly or in combination. Supplementary Table 5

Supplementary Table 6 Analysis of the Value of Information given by the Expected Value of Perfect Information. Supplementary Table 6

## Funding

This study was funded by the German Research Foundation (DFG), Bonn, Germany [grant number 458328858, 2021].

## Ethical considerations

This study received ethical clearance from the University of Bonn Center for Development Research (31c_23 Maureen Nabatanzi), Charité Universitätsmedizin Berlin (EA4/082/21), and the Uganda National Health Research Organization. Each participant voluntarily signed the consent form, which detailed the study's objectives, procedures, and data use. Participants' contributions were de-identified before data analysis and use.

## CRediT authorship contribution statement

**Maureen Nabatanzi:** Writing – review & editing, Writing – original draft, Visualization, Validation, Software, Methodology, Investigation, Formal analysis, Data curation, Conceptualization. **Selina L. Graff:** Writing – review & editing. **Peter Z. Sabakaki:** Writing – review & editing. **Teddy A. Tindyebwa:** Writing – review & editing. **Julius J. Lutwama:** Writing – review & editing, Supervision, Resources, Project administration, Funding acquisition, Conceptualization. **Innocent B. Rwego:** Writing – review & editing, Supervision, Resources, Project administration, Conceptualization. **Anthony M. Nsubuga:** Writing – review & editing, Supervision, Resources, Project administration, Funding acquisition, Conceptualization. **Sandra Junglen:** Writing – review & editing, Supervision, Resources, Project administration, Funding acquisition, Conceptualization. **Lisa Biber-Freudenberger:** Writing – review & editing, Validation, Supervision, Software, Resources, Project administration, Methodology, Funding acquisition, Conceptualization.

## Declaration of generative AI and AI-assisted technologies in the writing process

During the preparation of this work, the author used ChatGPT and Grammarly to correct grammar and improve the readability of the manuscript. After using these tools, the author reviewed and edited the content as needed and takes full responsibility for the content of the published article.

## Declaration of competing interest

The authors have declared that no competing interests exist.

## Data Availability

I have shared a link to my data in the manuscript
