# Supplementary material for: Modeling policy decisions to mitigate the risk of emerging arboviral diseases under ecological changes in Uganda: Proposing a one Health in all policies approach
Source: One Health. 2026 Apr 17;22:101414. doi: 10.1016/j.onehlt.2026.101414 (PMC13103579; doi:10.1016/j.onehlt.2026.101414)
Supplement: Supplementary Text 1 — Detailed methods. [file mmc1.docx]

**2. Methods**

**2.2.1 Problem framing and evidence synthesis**

We conducted a policy analysis to identify and compare the potential policy options that could reduce the risk of arboviral disease emergence [1]. We conducted an internet search for laws, policies, guidelines, strategies, and plans enacted or developed by the government of Uganda. We identified 13 documents with thematic connections to vectors, zoonoses, agriculture, wildlife, land use, and biodiversity within the context of arboviral diseases. We focused on the most recent main legal document that covered a theme. For example, the National Land Use Policy (2006 and 2013) were excluded in preference for the National Environment Act, No.5 (2019), which reflects updated land use and ecosystem protection strategies. Furthermore, documents that were not publicly accessible were excluded. During policy review, we identified disease preventive interventions and collated these into similar themes.

**2.2.2 Identification of decision objectives**

We purposively invited 20 stakeholder experts from government, academia, research, and civil society in the sectors of health, agriculture, environment, climate, and wildlife in Uganda (see Supplementary Table 1). The stakeholders were selected based on their knowledge, diversity, experience, and involvement in policy/program decision making [2-4]. We conducted two stakeholder workshops. In the first, we presented the policy analysis findings for stakeholders to refine. They also identified six additional policies for inclusion and agreed on four main policy scenarios for disease mitigation. This process informed the decision, i.e., the choice between two or more alternatives involving an irrevocable allocation of resources [5].

**2.2.3 Specification of alternative actions and valuation of their impacts**

During the second workshop, we divided stakeholders into interdisciplinary groups. Using a schematic summary of the intervention themes identified in the policy analysis, stakeholders discussed their desired objectives concerning the decision for each stakeholder. We aligned the objectives with the list of themes. This was followed by iterative plenary sessions until we generated consensus on 29 actions to be prioritised in the prevention of arboviral disease risk. Similar preventive actions were clustered into groups, forming four implementation packages [6]. All these were formulated into a directed acyclic graph (DAG) comprising directed arcs linking policy options to actions, implementation packages, and ultimately arbovirus risk [5].

**2.2.4 Evaluation of outcomes using Bayesian models**

Bayesian decision modeling applies Bayes' theorem to incorporate expert knowledge in probabilistic models [6]. We transformed the directed acyclic graph (DAG) from SDM into a Bayesian Network (BN), representing relationships between policy options (parent nodes) and preventive actions (child nodes). Conditional probabilities of implementing each preventive action, given each policy state (Yes/No), were elicited based on expert estimates of likely outcomes rather than precise values [5].

Participants underwent a calibration process to evaluate their uncertainty and reduce potential biases in probability estimation [2, 5]. Calibration involved rounds of trivia questions. For each question, participants estimated a value and its range of values for the upper and lower boundary of a 90% confidence interval, i.e. a range in which they were 90% certain the correct answer lay [5]. Per round, right answers and relative error were presented such that participants could weigh their uncertainty and discuss readjustments; this was done until participants refined their uncertainty judgements.

Using Microsoft Excel, the experts entered the prior probabilities for the likelihood of implementing each of the 29 preventive actions under each policy state. We considered equal weights for all expert responses, checked them for inconsistencies, aggregated them, and calculated median probabilities per preventive action using the R package “dplyr” [7].

Since it was impractical to elicit full conditional probability tables for implementation package nodes and the disease risk node, we instead computed conditional probabilities using a weighted scoring approach [5, 6, 8]. We first translated the implementation package probabilities into ordinal score categories (High, Medium, Low) [5, 6]. The weighting cut-offs assumed scores above 0.7 as better than average while below 0.4 were considered below average. For each policy option *j*, the implementation score for a package *g* (e.g., *Surveillance*) was computed as the average probability of implementation across all preventive actions *i* = 1, …, *n_g_* within that package (1):

| $S_{gj}= \left\{ \begin{aligned} 1, if \frac{1}{n_{g}}\sum_{i = 1}^{n_{g}} P_{ij}\geq0.7 \\ 0.5, if 0.4 \leq\frac{1}{n_{g}} \sum_{i = 1}^{n_{g}} P_{ij} < 0.7 \\ 0, if \frac{1}{n_{g}}\sum_{i = 1}^{n_{g}} P_{ij}< 0.4 \end{aligned} \right.$ | (1) |
| --- | --- |

Where:

- *S_gj_* is the score of the implementation package *g* under policy *j*
- *P_ij_*: Probability of preventive action *i* being implemented under policy *j*
- *n_g_*​: Total number of preventive actions in package *g*

Implementation package scores *S_gj​_* were classified into ordinal states as follows:

- High if *S_gj_* ≥ 0.7, numerical value = 1
- Medium if 0.4 ≤ *S_gj_* < 0.7, numerical value = 0.5
- Low if *S_gj_* < 0.4, numerical value = 0

These ordinal numerical values were subsequently used as input for downstream probabilistic inference in the Bayesian network. Arboviral disease risk score *R_j_* under each policy option *j*, was computed as the average of the four intermediate implementation packages—Surveillance, Biodiversity protection, Vector control, and Interdisciplinary actions—under that policy option (2):

| $R_{j}= \left\{ \begin{aligned} 1, if \frac{1}{4}\sum_{g = 1}^{4} S_{gj}\leq0.4 \\ 0.5, if 0.4 < \frac{1}{4} \sum_{g = 1}^{4} S_{gj} \leq0.7 \\ 0, if \frac{1}{4}\sum_{g = 1}^{4} S_{gj}> 0.7 \end{aligned} \right.$ | (2) |
| --- | --- |

Where:

1. *R_j_* is the arboviral disease risk score *R* under policy option *j*
2. *S_gj_* ​ is the score for implementation package *g* under policy option *j*

Risk scores were classified into ordinal states:

- Low, if score ≥ 0.7
- Medium, if 0.4 < score < 0.7
- High, if score ≤ 0.4

The conditional probabilities for the preventive actions and weighted score equations for the implementation packages and arboviral disease risk were entered in Netica version 7.01, which computed the BN [9].

- - 1. **Trade-off analysis to optimise decisions**

The Value of Information (VoI) analysis utilized Microsoft Excel. We calculated the Expected Value of Perfect Information (EVPI), which quantifies the benefit of eliminating uncertainty across all policy options. Based on participants’ preference for minimising arboviral disease risk, we assigned the highest utility to scenarios where high effort (OH policy) coincides with high risk, or no effort (DN policy) coincides with low risk. Intermediate utilities were assigned to moderate policy efforts and disease risk levels.

For each policy option *j*, the Expected Monetary Value (EMV) was calculated as the sum of the products of utility values and their corresponding posterior probabilities across disease risk states *t* (3):

| $\text{EMV}_{j}=\sum_{t=1}^{T} U_{jt}\cdot P_{t}$ | (3) |
| --- | --- |

Where:

- *U_jt_​* is the utility (e.g., benefit) of policy *j* under risk state *t*
- *P_t_*_​_ is the posterior probability of disease risk state *t*
- *T* is the number of risk states

The Expected Value with Perfect Information (EV with PI) *EV_PI_* was estimated as the sum of the products of the maximum utility value achievable for each risk state and policy option *max_j_(U_jt_)* and its corresponding probability *P_t_* (4):

| ${EV}_{PI}= \sum_{t=1}^{T} {max}_{j}\left( U_{jt} \right) \times P_{t}$ | (4) |
| --- | --- |

We then computed EVPI as the difference between the expected value with perfect information and the maximum EMV across all policy options max_j_(EMV_j_) (5):

| $EVPI = {EV}_{PI} - {max}_{j}\left( {EMV}_{j} \right)$ | (5) |
| --- | --- |

**References**

1. Control USCfD. Policy Analysis: USA Government; 2024 [updated September 27, 202413/07/2025]. Available from: <https://www.cdc.gov/polaris/php/cdc-policy-process/policy-analysis.html>.

2. Hubbard DW. How to Measure Anything: Finding the Value of Intangibles in Business. 3rd edition. ed. Newark: Wiley; 2014.

3. Shepherd K, Hubbard D, Fenton N, Claxton K, Luedeling E, de Leeuw J. Policy: Development goals should enable decision-making. Nature. 2015;523(7559):152-4. doi: 10.1038/523152a.

4. Freebairn L, Atkinson J-A, Kelly PM, McDonnell G, Rychetnik L. Decision makers’ experience of participatory dynamic simulation modelling: methods for public health policy. BMC Medical Informatics and Decision Making. 2018;18(1):131. doi: 10.1186/s12911-018-0707-6.

5. Whitney C SK, Luedeling E. . Decision analysis methods guide. Working Paper No. 275. Nairobi: 2018.

6. Whitney CW, Lanzanova D, Muchiri C, Shepherd KD, Rosenstock TS, Krawinkel M, et al. Probabilistic Decision Tools for Determining Impacts of Agricultural Development Policy on Household Nutrition. 2018;6(3):359-72. doi: <https://doi.org/10.1002/2017EF000765>.

7. Wickham H FR, Henry L, Müller K, Vaughan D. dplyr: A Grammar of Data Manipulation. R package version 1.1.4 ed2023.

8. Whitney C, Biber-Freudenberger L, Luedeling E. Decision analytical methods for assessing the efficacy of agroecology interventions. CABI Agriculture and Bioscience. 2023;4(1):11. doi: 10.1186/s43170-023-00151-9.

9. Corp NS. Netica "7.01" 64 Bit For MS Windows 7 to 11. 2023. p. Netica is a complete software package to work with Bayesian belief networks, decision nets and influence diagrams.
