# Supplementary material for: Modeling policy decisions to mitigate the risk of emerging arboviral diseases under ecological changes in Uganda: Proposing a one Health in all policies approach
Source: One Health. 2026 Apr 17;22:101414. doi: 10.1016/j.onehlt.2026.101414 (PMC13103579; doi:10.1016/j.onehlt.2026.101414)
Supplement: Supplementary Table 1 — Anonymised list of experts who participated in the workshops. [file mmc2.docx]

**Supplementary Table 1: Anonymised list of experts who participated in the workshops**

| **Organisation** | **Position** | **Sector** |
| --- | --- | --- |
| Ministry of Health | Epidemiologist | Human health |
| Ministry of Agriculture Animal Industries and Fisheries | Senior Veterinary Officer | Animal health |
| Ministry of Agriculture Animal Industries and Fisheries | Senior Veterinary Officer | Animal health |
| Conservation Through Public Health | Chief Veterinary Technician | Wildlife |
| Ministry of Water and Environment | Environment Officer | Environment |
| Uganda National Meteorological Authority | Principal Meterologist | Climate |
| Ministry of Water and Environment | Environment Officer | Environment |
| Makerere School of Public Health | Lecturer | Education |
| Makerere University Walter Reed Research Program | Virologist | Research |
| Uganda Virus Research Institute | Biostatistician | Human health |
| Makerere University Walter Reed Research Program | Virologist | Research |
| Uganda National Meteorological Authority | Meteorologist | Climate |
| Infectious Diseases institute | Biostatistician | Human health |
| Makerere University Walterreed Research Program | Virologist | Health |
| Uganda Virus Research Institute | One Health Project Manager | One Helalth |
| District Local Government | District Veterinary Officer | Local government |
| Ministry of Health | Research Officer | Human health |
| Ministry of Water and Environment | Environment Officer | Environment |
| Ministry of Health | Biostatistician | Health information |
| National Environment Management Authority | Environment Inspector | Environment |
