# Supplementary material for: Modeling policy decisions to mitigate the risk of emerging arboviral diseases under ecological changes in Uganda: Proposing a one Health in all policies approach
Source: One Health. 2026 Apr 17;22:101414. doi: 10.1016/j.onehlt.2026.101414 (PMC13103579; doi:10.1016/j.onehlt.2026.101414)
Supplement: Supplementary Table 2 — Summary of policy documents reviewed and identified intervention themes and gaps linked to arboviral disease risk mitigation. [file mmc3.docx]

**Supplementary Table 2: Summary of policy documents reviewed and identified intervention themes and gaps linked to arboviral disease risk mitigation**

| **Act/Policy/Guideline/Strategy** | **Sector** | **Main objective** | **Themes (n = 162)** | **Notable gaps** | **Reference** |
| --- | --- | --- | --- | --- | --- |
| Integrated Disease Surveillance and Response guidelines, 3rd edition (2021) | Ministry of Health | A general reference for public health surveillance activities across all levels to improve early detection and preparedness for outbreak response | 24 | Limited guidance on sentinel, genomic and animal surveillance for arboviruses. Inadequate provisions for ecological investigations and climate change. Surveillance goals for the priority arboviral diseases mainly syndromic leaving out pre-epidemic emerging infections. Guidance on reservoir or risk assessments is vague. Is targeting priority or International Health Regulations (IHR) notifiable diseases, not emerging pathogens. | [1] |
| Ministry of Health Strategic Plan (2020/21-2024/25) | Ministry of Health | Outlines the mandate of the MoH in governance, leadership and coordination of health programs. Presents strategies for strengthening disease surveillance, epidemic control and disaster preparedness and response at national and sub-national levels. Advocates for "Health in All Policies", a collaborative approach that integrates and articulates health considerations into policymaking across sectors to improve the health of all communities and people. | 19 | Environmental Impact Assessments (EIA) are only addressed in the context of health facility infrastructural construction. Environmental issues planned for only in context of WASH. The mechanism of implementing proposed intersectoral collaboration to mitigate environmental and climatic determinants of climate sensitive diseases is not articulated. Implications of biodiversity loss and land use changes on disease emergence not addressed. | [2] |
| National Biodiversity Strategy and Action Plan II (2015-2025) | Ministry of Water and Environment, National Environment Management Authority | To enhance biodiversity conservation, management and sustainable utilisation and fair sharing of the benefits. | 14 | Implications of biodiversity loss, and land use changes on human disease emergence are not addressed. Limited linkage with human health. | [3] |
| National Action Plan for Health Security (2024/25-2028/29) | Multiple Sectors and non-government stakeholders | To strengthen the country’s capacity to prevent, detect and respond to public health threats. To strengthen the collaboration and coordination mechanism for the National Action Plan for Health Security (NAPHS) implementation  through application of multi-sectoral and one health approaches. To map and align existing and potential domestic and external financing to support NAPHS  implementation. | 13 | Strengthening laboratory capacity for genomic surveillance focuses on routine disease surveillance and outbreak response, excluding pre-epidemic monitoring. Climate change mitigation through trainings, tree planting and green energy use at health facilities, but not how it can be applied to disease prevention e.g. Through the integration of climatic data in surveillance. | [4] |
| Framework for Multi-hazard Preparedness and response to Public Health Emergencies (2023) | Ministry of Health | Harmonise processes and guide stakeholders on strengthening public health emergency preparedness, response and recovery. The framework focuses on addressing health risks associated with all hazards using a multi-sectoral approach. | 13 | The framework focuses on the detection and response aspects of preparedness and largely neglects the pre-epidemic monitoring of potential hazards like emerging pathogens that don't have specific contingency plans. Also, pre-epidemic activities like risk assessments, reservoir investigation, climate and vector monitoring aren't addressed. Role of the environment sector in controlling environmental determinants of public health emergencies beyond water and sanitation is not addressed. | [5] |
| Infection Prevention and Control Plan for the Agricultural Sector (2023 - 2025) | Ministry of Agriculture, Animal Fisheries and Industry | Strengthen plant and animal health systems through infection prevention at the farm level as a cost-effective way of reducing plant and animal pests and diseases, as well as the occurrence of antimicrobial resistance (AMR). | 13 |  | [6] |
| Uganda Wildlife Act 17 (2019) | Ministry of Tourism, Wildlife and Antiquities, Uganda Wildlife Authority | To provide for the conservation and sustainable management of wildlife; to strengthen wildlife conservation and management; to continue the existence of the Uganda Wildlife Authority; to streamline the roles and responsibilities of institutions involved in wildlife conservation and management; to continue the existence of the Wildlife Fund; to repeal the Uganda Wildlife Act, Cap. 200, and related matters. | 10 | Linkage with public health, livestock health and or zoonotic diseases is missing | [7] |
| Uganda National Climate Change Policy (2015) | Ministry of Water and Environment | Ensure that all stakeholders address climate change impacts and their causes through appropriate measures while promoting sustainable development and a green economy. | 8 |  | [8] |
| Uganda Wildlife Policy (2014) | Ministry of Tourism, Wildlife and Antiquities, Uganda Wildlife Authority | To conserve the wildlife resources of Uganda in a manner that contributes to the sustainable development of the nation and the well-being of its people. | 8 | Linkage with animal and human health sectors is implied but not articulated in terms of zoonotic disease management | [9] |
| Climate Change Health National Adaptation Plan (2025-2030) | Ministry of Health | Framework for climate change adaptation actions for the health sector | 7 |  | [10] |
| National Environment Management Policy (1995) | Ministry of Water and Environment | Framework to guide these actions and is the cornerstone of the country's commitment to social and economic development that is environmentally sustainable, and which will bring the benefits of a better life to all Ugandans. Transform the existing environmental management system to establish an integrated and multi-sectoral approach to resource planning and management by creating a National Environment Management Authority (NEMA) | 6 | Implications of biodiversity loss, and land use changes on human disease emergence are not addressed. Limited linkage with human health. | [11] |
| Case definitions for priority animal diseases in Uganda (2023) | Ministry of Agriculture, Animal Fisheries and Industry | Intended to guide veterinarians and veterinary paraprofessionals to quickly  detect, investigate, report, and respond appropriately to animal diseases to prevent death and/or further spread. | 5 | Limited guidance on sentinel and genomic surveillance for diseases. Inadequate provisions for ecological investigations and climate change, and how those data can guide surveillance. Surveillance goals for the priority animal diseases focus on the identification of suspected cases, i.e., it is mainly syndromic, which leaves out pre-epidemic emerging infections. | [12] |
| National Environment Act Cap 181 (2019) | National Environment Management Authority | The National Environment Act Cap 181 provides for the overall management, coordination and monitoring of environment management and conservation in Uganda. It provides for the protection and conservation of natural resources in Uganda as well as promotion of international cooperation in the field of the environment. | 4 | Recognises some environmental hazards that can affect human health, but not in terms of disease emergence | [13] |
| Animal Diseases (Amendment) Act 26 of 2006 | Ministry of Agriculture, Animal Fisheries and Industry | This Act makes provision with respect to measures to control diseases affecting animals. | 4 | Linkage with wildlife and human health sectors implied but not articulated in terms of zoonotic disease management. | [14] |
| Public Health (Amendment) Act (2023) | Ministry of Health | Amends the Public Health Act by updating the law regarding the preservation of public health | 4 | Limited linkage with the environmental sector as per the determinants of epidemic-prone diseases | [15] |
| Veterinary Practitioner's Act 2023: Section 22 (C) | Ministry of Agriculture, Animal Fisheries and Industry | An Act to provide for the regulation of veterinary practice by providing for the training, registration and licensing of veterinary professionals and veterinary para-professionals; to provide for matters incidental to the conduct of veterinary professionals and para-professionals in relation to animal health, production, welfare, public health and biosecurity services; to repeal the Veterinary Surgeons Act, Cap. 277. | 4 | Limited provisions for the implementation of veterinary public health and environmental health | [16] |
| National One Health Strategic Plan (2018-2022) | Ministry of Health, MAAIF and MWE | Build resilient, sustainable systems to prevent and respond to zoonotic diseases and address Anti-Microbial Resistance (AMR) and biosecurity. | 4 | Focuses on priority zoonotic diseases and less on emerging zoonotic threats. Environmental and climatic determinants of zoonotic diseases are unaddressed. | [17] |
| National Agriculture Policy (2013) | Ministry of Agriculture, Animal Fisheries and Industry | To achieve food and nutrition security and improve household incomes through coordinated interventions that focus on enhancing sustainable agricultural productivity and value addition, providing employment opportunities, and promoting domestic and international trade. | 1 | Vector control guidance is biased towards tick vectors. Limited guidance on animal disease surveillance. Limited linkage with health sector plans in terms of zoonotic diseases | [18] |
| National Climate Change Act, Cap 2021 (2021) | Ministry of Water and Environment, Uganda National Meteorological Authority | The Act provides for climate change response measures, participation in climate change mechanisms, measuring of emissions, reporting and verification of information, institutional arrangements for coordinating and implementing climate change response measures, financing for climate change and related matters. | 1 | Limited linkage to climatic determinants of disease emergence | [19] |

**References**

1. Health Mo. National Technical Guidelines for Integrated Disease Surveillance and Response. Third ed. Kampala, Uganda2021.

2. Health Mo. Ministry of Health Strategic Plan (2020/21-2024/25). Kampala, Uganda: Government of Uganda; 2020.

3. Uganda Go. National Biodiversity Strategy and Action Plan II (NBSAP II) 2015-2025. Kampala, Uganda: National Environment Management Authority; 2016.

4. Uganda Go. National Action Plan for Health Security III (2024/25-2028/29). Kampala, Uganda2024.

5. Uganda Go. Framework for Multi-hazard Preparedness and response to Public Health Emergencies (2023) Kampala, Uganda2023. Available from: <https://www.health.go.ug/wp-content/uploads/2024/05/Multihazard-Preparedness-and-Response-Framework-for-Public-Health-Emergencies-final-draft-06th-October-2023-Approved-by-Minister.pdf>.

6. Uganda Go. Infection Prevention and Control Plan for the Agricultural Sector (2023 - 2025). Kampala, Uganda2022.

7. Uganda Go. Uganda Wildlife Act 17 (2019). Kampala, Uganda2019.

8. Uganda Go. Uganda National Climate Change Policy (2015). Kampala, Uganda: Ministry of Water and Environment of Uganda; 2015.

9. Uganda Go. Uganda Wildlife Policy 2014. In: Ministry of Tourism WaA, editor. Kampala, Uganda2014.

10. Uganda Go. Climate Change Health National Adaptation Plan (2025-2030). Kampala, Uganda2024.

11. National Environment Management Policy (1995), (1995).

12. Uganda Go. Case definitions for priority animal diseases in Uganda (2023). In: Ministry of Agriculture AFaI, editor. Kampala, Uganda2023.

13. The National Environment Act, 2019, (2019).

14. Animal Diseases (Amendment) Act 2006, (2006).

15. Public Health (Amendment) Act (2023) (2023).

16. Uganda Go. Veterinary Practitioner's Act 2023: Section 22 (C) Kampala, Uganda2023. Available from: <https://bills.parliament.ug/attachments/Veterinary%20Practitioners%20Act,%202023.pdf>.

17. Uganda Go. National One Health Strategic Plan (2018-2022). In: Ministry of Health MoA, Animal Industry and Fisheries, Uganda Wildlife Authority and Ministry of Water and Environment, editor. Kampala, Uganda2018.

18. National Agriculture Policy (2013), (2013).

19. National Climate Change Act, Cap 2021, (2021).
