# Supplementary material for: Modeling policy decisions to mitigate the risk of emerging arboviral diseases under ecological changes in Uganda: Proposing a one Health in all policies approach
Source: One Health. 2026 Apr 17;22:101414. doi: 10.1016/j.onehlt.2026.101414 (PMC13103579; doi:10.1016/j.onehlt.2026.101414)
Supplement: Supplementary Table 3 — Intervention themes identified in the policy analysis and the number of times they appeared. [file mmc4.docx]

**Supplementary Table 3: Intervention themes identified in the policy analysis and the number of times they appeared**

| **Intervention themes** | **Count** |
| --- | --- |
| Community sensitive wildlife/biodiversity | 9 |
| One Health response | 8 |
| Livestock surveillance | 6 |
| Report livestock diseases | 6 |
| Climate change mitigation trainings | 5 |
| Interoperable intersectoral surveillance data systems | 5 |
| Operationalize intersectoral zoonotic data management | 5 |
| Restore ecosystems and wildlife protected areas | 5 |
| Sustainable land use balancing biodiversity protection, agriculture, economic activities and urbanization | 5 |
| Climate vulnerability and risk assessment | 4 |
| Control livestock diseases | 4 |
| Manage protected areas | 4 |
| Robust One Health implementation | 4 |
| Strengthen public health surveillance and response to epidemics | 4 |
| Zoonotic risk assessment | 4 |
| Afforestation | 3 |
| Combat wildlife poaching/trade | 3 |
| Intersectoral contigency planning | 3 |
| Mitigate human-wildlife conflicts | 3 |
| Mosquito net use | 3 |
| Vector surveillance | 3 |
| Agroforestry | 2 |
| Animal laboratory diagnostic capacity | 2 |
| Climate change mitigation | 2 |
| Control wildlife diseases | 2 |
| EIAs for development projects | 2 |
| Early detection human | 2 |
| Enhance early-warning for climate-sensitive diseases | 2 |
| Laboratory diagnosis human | 2 |
| Laboratory diagnosis of livestock diseases | 2 |
| Limit human-wildlife-livestock contact | 2 |
| Public health surveillance trainings | 2 |
| Remove larval habitat | 2 |
| Report risky events among animals | 2 |
| Risk assessments of suspected events | 2 |
| Robust One Health implementation | 2 |
| Strengthen district public health surveillance | 2 |
| Surveillance data management livestock | 2 |
| Surveillance livestock | 2 |
| Sustainable forest management | 2 |
| Wildlife surveillance | 2 |
| District One Health capacity | 1 |
| Early detection livestock | 1 |
| Early reporting wildlife | 1 |
| Indoor Residual Spraying | 1 |
| Integrate climate data in public health surveillance | 1 |
| Intersectoral climate-health coordination | 1 |
| Intersectoral collaboration in public health | 1 |
| Manage development projects in wildlife conservation areas | 1 |
| Pre-epidemic risk assessment | 1 |
| Prevent epidemics | 1 |
| Prevent wildlife diseases | 1 |
| Public health surveillance data management | 1 |
| Reduce human wildlife interaction | 1 |
| Reporting human suspected cases and risky events | 1 |
| Risk communication for One Health | 1 |
| Risk communication for animal diseases and vector control | 1 |
| Risk communication for public health events | 1 |
| Risk communication of climate and health information | 1 |
| Risk communication on wildlife conservation | 1 |
| Screening at Points of Entry | 1 |
| Sentinel surveillance | 1 |
| Strengthen animal surveillance data capacities | 1 |
| Train community on public health vector control | 1 |
| Train farmers and communities | 1 |
| Vector control livestock | 1 |
| Wetland protection | 1 |
| Total | 162 |
