# Supplementary material for: Modeling policy decisions to mitigate the risk of emerging arboviral diseases under ecological changes in Uganda: Proposing a one Health in all policies approach
Source: One Health. 2026 Apr 17;22:101414. doi: 10.1016/j.onehlt.2026.101414 (PMC13103579; doi:10.1016/j.onehlt.2026.101414)
Supplement: Supplementary Table 4 — Preventive actions and implementation packages. [file mmc5.docx]

**Supplementary Table 4: Preventive actions and implementation packages**

| **Preventive action (n = 29)** | **Implementation packages (n = 4)** |
| --- | --- |
| Train community and farmers to identify and report | Surveillance |
| Surveillance by community health, wildlife and extension workers |  |
| Screen livestock and humans at Points of Entry (PoE) |  |
| Household surveillance |  |
| Health facility sentinel surveillance |  |
| Animal regional surveillance laboratories and sites |  |
| Wildlife surveillance |  |
| Sentinel livestock herd surveillance |  |
| Forest conservation | Biodiversity protection |
| Wetland conservation |  |
| Limit agriculture in protected areas |  |
| Community wildlife biodiversity programs |  |
| Manage protected areas |  |
| Prevent wildlife poaching |  |
| Wetlands as flood water buffers | Vector control |
| Mosquito net use |  |
| Indoor residual spraying |  |
| Remove vector breeding sites |  |
| Vector surveillance |  |
| Vector screening at PoE |  |
| Vector sensitive urban planning |  |
| Train District One Health Teams | Interdisciplinary actions |
| Intersectoral zoonotic and climate data sharing |  |
| Biodiversity assessments at risky sites |  |
| Mosquito animal and human sampling at risky sites |  |
| Climate vulnerability assessment |  |
| Regulate infrastructural and mineral projects |  |
| Risk communication |  |
| Integrate arbovirus risk in environmental impact assessments |  |
