# Supplementary material for: Modeling policy decisions to mitigate the risk of emerging arboviral diseases under ecological changes in Uganda: Proposing a one Health in all policies approach
Source: One Health. 2026 Apr 17;22:101414. doi: 10.1016/j.onehlt.2026.101414 (PMC13103579; doi:10.1016/j.onehlt.2026.101414)
Supplement: Supplementary Table 5 — Percentage probability of arboviral disease risk under alternate policies given that the scenario states “High” of the implementation packages are scaled up to 100% singly or in combination. [file mmc6.docx]

**Supplementary Table 5: Percentage probability of arboviral disease risk under alternate policies given that the scenario states “High” of the implementation packages are scaled up to 100% singly or in combination**

| **Policy option** | **Implementation package scenarios** | **Probability of arboviral risk = High** | **Probability of arboviral risk = Medium** | **Probability of arboviral risk = Low** |
| --- | --- | --- | --- | --- |
| **Do nothing** | Surveillance | 74.3 | 25.1 | 0.57 |
|  | Biodiversity protection | 85.8 | 14 | 0.14 |
|  | Vector control | 86 | 13.9 | 0.15 |
|  | Interdisciplinary | 77 | 22.6 | 0.38 |
|  | Surveillance + Biodiversity protection | 0 | 90.6 | 9.42 |
|  | Surveillance + Vector control | 0 | 90.6 | 9.44 |
|  | Surveillance + Interdisciplinary | 0 | 82.1 | 17.9 |
|  | Biodiversity protection + Vector control | 0 | 96.9 | 3.06 |
|  | Biodiversity protection + Interdisciplinary | 0 | 92.9 | 7.07 |
|  | Vector control + Interdisciplinary | 0 | 92.8 | 7.21 |
|  | Surveillance + Biodiversity protection + Vector control | 0 | 0 | 100 |
|  | Surveillance + Vector control + Interdisciplinary | 0 | 0 | 100 |
| **Biodiversity conservation** | Surveillance | 5.24 | 54 | 40.7 |
|  | Biodiversity protection | 17.2 | 61.9 | 20.9 |
|  | Vector control | 3.87 | 46.9 | 49.3 |
|  | Interdisciplinary | 7.5 | 59.4 | 33.1 |
|  | Surveillance + Biodiversity protection | 0 | 41.4 | 58.6 |
|  | Surveillance + Vector control | 0 | 11.1 | 88.9 |
|  | Surveillance + Interdisciplinary | 0 | 21.1 | 78.9 |
|  | Biodiversity protection + Vector control | 0 | 32.7 | 67.3 |
|  | Biodiversity protection + Interdisciplinary | 0 | 50.9 | 49.1 |
|  | Vector control + Interdisciplinary | 0 | 15.7 | 84.3 |
|  | Surveillance + Biodiversity protection + Vector control | 0 | 0 | 100 |
|  | Surveillance + Vector control + Interdisciplinary | 0 | 0 | 100 |
| **Human and animal health** | Surveillance | 9.84 | 57.9 | 32.3 |
|  | Biodiversity protection | 5.3 | 48.8 | 45.9 |
|  | Vector control | 8.64 | 55.4 | 36 |
|  | Interdisciplinary | 6.35 | 54.1 | 39.6 |
|  | Surveillance + Biodiversity protection | 0 | 24.4 | 75.6 |
|  | Surveillance + Vector control | 0 | 35.5 | 64.5 |
|  | Surveillance + Interdisciplinary | 0 | 29.7 | 70.3 |
|  | Biodiversity protection + Vector control | 0 | 21.5 | 78.5 |
|  | Biodiversity protection + Interdisciplinary | 0 | 16.5 | 83.5 |
|  | Vector control + Interdisciplinary | 0 | 28.7 | 71.3 |
|  | Surveillance + Biodiversity protection + Vector control | 0 | 0 | 100 |
|  | Surveillance + Vector control + Interdisciplinary | 0 | 0 | 100 |
| **One Health** | Surveillance | 0.1 | 9.3 | 90.6 |
|  | Biodiversity protection | 0.14 | 10.3 | 89.6 |
|  | Vector control | 0.061 | 6.66 | 93.3 |
|  | Interdisciplinary | 0.045 | 6.47 | 93.5 |
|  | Surveillance + Biodiversity protection | 0 | 3.44 | 96.6 |
|  | Surveillance + Vector control | 0 | 1.71 | 98.3 |
|  | Surveillance + Interdisciplinary | 0 | 1.37 | 98.6 |
|  | Biodiversity protection + Vector control | 0 | 2.14 | 97.9 |
|  | Biodiversity protection + Interdisciplinary | 0 | 1.79 | 98.2 |
|  | Vector control + Interdisciplinary | 0 | 1.12 | 98.9 |
|  | Surveillance + Biodiversity protection + Vector control | 0 | 0 | 100 |
|  | Surveillance + Vector control + Interdisciplinary | 0 | 0 | 100 |
