# Supplementary material for: Modeling policy decisions to mitigate the risk of emerging arboviral diseases under ecological changes in Uganda: Proposing a one Health in all policies approach
Source: One Health. 2026 Apr 17;22:101414. doi: 10.1016/j.onehlt.2026.101414 (PMC13103579; doi:10.1016/j.onehlt.2026.101414)
Supplement: Supplementary Table 6 — Analysis of the Value of Information given by the Expected Value of Perfect Information. [file mmc7.docx]

**Supplementary Table 6:** **Analysis of the Value of Information given by the Expected Value of Perfect Information**

| **Probabilities of policy scenarios and risk** | **Risk = High** | **Risk = Medium** | **Risk = Low** | **Expected Monetary Value** | **EV with PI** | **EVPI** |
| --- | --- | --- | --- | --- | --- | --- |
| Do Nothing | 10 | 20 | 90 |  | | |
| Probability P(Risk\|B) | 0.319 | 0.546 | 0.135 |  | | |
| Biodiversity conservation, **B** | 30 | 40 | 60 | **39.5** | **79.1** | **17.1** |
| Probability P(Risk\|H) | 0.304 | 0.535 | 0.161 |  | | |
| Human and animal health, **H** | 50 | 60 | 70 | **58.6** | **79.3** | **17.3** |
| Probability P(Risk\|OH) | 0.0069 | 0.176 | 0.817 |  | | |
| One Health, **OH** | 90 | 70 | 60 | **62.0** | **86.5** | **24.5** |
